# Toxicity Effects of Combined Mixtures of BDE-47 and Nickel on the Microalgae *Phaeodactylum tricornutum* (Bacillariophyceae)

**DOI:** 10.3390/toxics10050211

**Published:** 2022-04-22

**Authors:** Xiaolai Shi, Ruoyu Guo, Douding Lu, Pengbin Wang, Xinfeng Dai

**Affiliations:** Key Laboratory of Marine Ecosystem Dynamics, Second Institute of Oceanography, Ministry of Natural Resources, 36 Baochubei Road, Hangzhou 310012, China; shxl7509@sio.org.cn (X.S.); dinoflagellate@sio.org.cn (R.G.); doudinglu@sio.org.cn (D.L.)

**Keywords:** toxicity, physiological, transcriptome, the combination of BDE-47 and nickel, *Phaeodactylum tricornutum*

## Abstract

Nickel and 2,2’,4,4’-tetrabromodiphenyl ether (BDE-47) are two environmental pollutants commonly and simultaneously present in aquatic systems. Nickel and BDE-47 are individually toxic to various aquatic organisms. However, their toxicity mechanisms are species-dependent, and the toxic effects of combined mixtures of BDE-47 and nickel have not yet been investigated. The present study investigated the toxic effects of combined mixtures of BDE-47 and nickel in the diatom *Phaeodactylum tricornutum*. BDE-47 and nickel mixtures significantly decreased cell abundance and photosynthetic efficiency, while these cells’ reactive oxygen species (ROS) production significantly increased. The EC_50_-72 h for BDE-47 and mixtures of BDE-47 and nickel were 16.46 ± 0.93 and 1.35 ± 0.06 mg/L, respectively. Thus, combined mixtures of the two pollutants enhance their toxic effects. Interactions between BDE-47 and nickel were evaluated, revealing synergistic interactions that contributed to toxicity in *P. tricornutum*. Moreover, transcriptomic analyses revealed photosynthesis, nitrogen metabolism, the biosynthesis of amino acids, the biosynthesis of secondary metabolites, oxoacid metabolism, organic acid metabolism, carboxylic acid metabolism, and oxidation-reduction processes were considerably affected by the mixtures. This study provides evidence for the mechanisms of toxicity from combined BDE-47 and nickel exposure while also improving our understanding of the ecological risks of toxic chemicals on microalgae.

## 1. Introduction

Increasing concentrations of chemical pollutants, including heavy metals and organic pollutants from anthropogenic activities, have significantly affected aquatic ecosystems by inhibiting cellular function and growth of aquatic organisms and even killing organisms [1,2,3]. Numerous ecological risk assessments have been conducted for various individual chemical pollutants [4,5]. However, the need to assess risks for combinations of chemicals has become apparent since organisms are exposed to mixtures of chemical pollutants in natural environments rather than individual compounds, especially in aquatic ecosystems [6,7,8].

Microalgae are major primary producers in aquatic ecosystems and biospheres. Microalgae can accumulate chemical pollutants in aquatic ecosystems and transfer them to higher trophic levels [9]. In addition, they are also sensitive to certain chemical pollutants, with many pollutants altering microalgal morphology, physiology, and genetics, thereby affecting their functions in aquatic ecosystems [9]. Consequently, microalgae are considered important models for assessing ecotoxicity, with numerous toxicity assessments previously published for microalgae [10,11,12].

Heavy metals and organic pollutants are two of the major toxic pollutants present. Nickel is a heavy metal used in diverse metallurgical, electronic, chemical, and food industries. The prevalence of nickel-based products has led to the inevitable pollution of the environment, with concentrations reaching 74.56 mg/kg in coastal surface sediments [13]. Nickel is also a micronutrient essential for some microalgal enzymes, such as urease in the diatom *Phaeodactylum tricornutum* [14]. Nevertheless, excess nickel can inhibit photosynthesis and decrease protein, carbohydrate, and lipid concentrations in microalgae [10,15]. The toxic effects of nickel on *P. tricornutum* have recently been investigated, revealing that nickel can inhibit *P. tricornutum* photosynthesis and induce reactive oxygen species (ROS) production [16]. Polybrominated diphenyl ethers (PBDEs) are persistent organic pollutants and flame retardants that have been used in commercial products since the 1960s [17]. PBDE pollutants are globally distributed in aquatic ecosystems and have been extensively detected in biological samples [2,18,19,20]. Indeed, PBDEs in coastal waters have been detected at a concentration of 65.5 ng/L in the Bohai Sea of China [21]. However, their concentrations are generally higher in coastal sediments than in open waters, as high as 4212 (ng/g dw) [22]. In most areas, decabromodiphenyl ether (BDE-209) and 2,2’,4,4’-tetrabromodiphenyl ether (BDE-47) are the predominant types of identified PBDEs [21,22]. The toxicity of BDE-47 toward microalgae has been evaluated in the algae *Skeletonema costatum*, *Thalassiosira pseudonana*, *Phaeodactylum tricornutum*, *Platymonas subcordiformis*, *Alexandrium minutum*, and *Dunaliella salina* [23,24,25]. These studies have shown that BDE-47 can inhibit photosynthetic efficiency, arrest cell division, and induce H_2_O_2_ production in microalgal cells [25,26,27,28]. The toxic effects of nickel and BDE-47 have been identified in several microalgae species, although the toxicity mechanisms vary [10,26,27,28,29]. In *P. tricornutum*, BDE-47 can damage chloroplasts, reduce the oxygen evolution rate, alter the performance of photosystems, and stimulate ROS production [24]. Nevertheless, studies of the combined toxicity of nickel and BDE-47 in microalgae remain limited.

*P. tricornutum* is a cosmopolitan marine diatom species widely used to study ecotoxicity [30], and its complete genome sequence has been published [31]. Moreover, the toxicity mechanisms of nickel on *P. tricornutum* have been recently reported [16]. Furthermore, the toxic effects of BDE-47 have also been recently evaluated for *P. tricornutum* based on photosynthesis-related parameters [24]. This study investigated the combined toxicity effects of BDE-47 and nickel on P. tricornutum via physiological and transcriptomic responses. At the same time, the interaction patterns of BDE-47 and nickel in *P. tricornutum* were also measured. This study provides additional understanding of the toxic effects of BDE-47 and nickel mixtures on *P. tricornutum.*

## 2. Materials and Methods

### 2.1. Cell Cultures and Chemical Treatments

Axenic algal cultures of the diatom *P. tricornutum* CCMP2561 were obtained from the National Center of Marine Algae and Microbiota (USA) and cultured with an f/2 medium at 20 °C (12:12 h light-dark cycles with 65 μmol photons/m^2^/s). The chemical toxicity studies used exponential growth phase cultures with an initial cell density of 5.2 × 10^5^ cells. To evaluate the influence of chemical pollutants on cultures, a gradient of individual BDE-47 (Dr. Ehrenstorfer GmbH, Germany), individual nickel (NiCl_2_·6H_2_O, Sigma-Aldrich, Louis, MO, USA), and mixtures of the compounds were added to *P. tricornutum* cultures (concentrations shown in Table S1), followed by harvesting cells at 24, 48, 72, and 96 h to assess cell abundance and photosynthetic efficiency. Each 200 mL of *P. tricornutum* cultures was maintained in glass flasks, and all the flasks were maintained in the same incubator. 

The actual concentrations of nickel and BDE-47 were verified with ICP-MS (inductively coupled plasma-mass spectrometry) and GC-MS (gas chromatograph-mass spectrometry), respectively. Moreover, relationships among the actual nickel concentrations and BDE-47 in mixtures were assessed with regression analysis (Table S1). BDE-47 was dissolved in dimethyl sulfoxide (DMSO, CNW, China), and untreated DMSO-amended cultures were used as controls. All treatments were performed in triplicate.

### 2.2. Physiological Parameter Analysis

Cell density, photosynthetic efficiency, and reactive oxidative species (ROS) levels were measured to evaluate the physiological responses of *P. tricornutum* to chemical exposure. *P. tricornutum* cells were fixed with glutaraldehyde (Sigma, Louis, MO, USA) and counted using a Countstar automated algae counter (Countstar algae, Shanghai, China). Photosynthetic efficiency (*F*v/*F*m) was directly determined using a pulse amplitude-modulated fluorometer (Water-PAM fluorometer, Walz, Germany) after adapting cultures to the dark. ROS production was determined by measuring fluorescence density. *P. tricornutum* cells were stained with 2’,7’-Dichlorodihydrofluorescein diacetate (DCFH-DA) according to the manufacturer’s instructions (Nanjing Jiancheng Bioengineering Institute, Nanjing, China). At the same time, ROS fluorescence density was measured using a fluorospectrophotometer (LS45 Fluorescence Spectrometer, PerkinElmer, Llantrisant, UK) with an excitation and emission wavelength of 515 nm and 550 nm, respectively. A sampling of the microalgae was performed in triplicate throughout the experiments.

### 2.3. Median Effective Concentrations (EC_50_)

The concentrations of BDE-47 and nickel/BDE-47 mixtures that induced a 50% reduction in *P. tricornutum* biomass after 72 h of exposure were calculated according to the Organization for Economic Cooperation and Development (OECD) guidelines [32] by estimating cell numbers. EC_50_-72 h values were estimated with a sigmoidal curve and the OriginPro 2018 software program (OriginLab Corporation, Northampton, MA, USA).

### 2.4. Statistical Analyses

One-way analysis of variance (ANOVA) tests with a subsequent Games–Howell test evaluated differences in parameter distributions. Levene’s tests were used to investigate homoscedasticity (Table S2). Data are presented as means ± standard deviations (SD), and statistical significance was determined at *p* < 0.05. The statistical results are shown in Tables S3–S5.

To assess the effects of the mixed treatment, EC_50_-72 h values were obtained from each compound, and the mixture treatments were used. Concentration addition (CA) and independent addition (IA) models were used to evaluate the interaction patterns of nickel and BED-47 mixtures, followed by an analysis with the MIXTOX tool [33]. Calculation details were described in a previous study [33]. These models evaluate putative interaction patterns for combinations of chemicals. CA models are generally used to evaluate combinations of chemicals that share the same mechanism of action. In contrast, IA models are generally used for those that exhibit different mechanisms of action. Nevertheless, it is difficult to confirm the model that should be used in many cases. The MIXTOX model can be used with the additional parameter ‘*a*’ to evaluate potential interaction patterns [34,35]. Briefly, the ‘*a*’ parameter was used to evaluate the synergism or antagonism of two chemical interactions, wherein *a* < 0 indicates synergism and *a* > 0 indicates antagonism.

### 2.5. Transcriptomic Response Analysis

An expected median concentration of 2.4 mg/L for pollutant mixtures was selected for transcriptomic analyses according to the EC_50_-48 and EC_50_-72 h values. The *P. tricornutum* cells were harvested at 48 and 72 h for subsequent transcriptome analysis. Specifically, 200 mL of *P. tricornutum* cultures were harvested, centrifuged, and stored at −80 °C until subsequent RNA extraction. Total RNA was isolated using the TRIzol reagent (Invitrogen, Carlsbad, CA, USA) according to the manufacturer’s instructions, followed by an evaluation of RNA integrity and quality with the RNA Nano 6000 Assay Kit for the Bioanalyzer 2100 system (Agilent Technologies, Santa Clara, CA, USA). mRNAs were purified to construct cDNA libraries for sequencing conducted at Novogene (Beijing, China) on the Illumina NovaSeq platform (Illumina Inc., San Diego, CA, USA). According to the manufacturer’s instructions, cDNA libraries were constructed with the NEBNext Ultra RNA Library Prep Kit for Illumina (New England BioLabs, USA).

Raw sequence reads were processed to obtain clean reads by removing adapters, N bases, and low-quality reads. Clean data were mapped to the *P. tricornutum* CCAP 1055/1 reference genome retrieved from the NCBI database. The reference genome index was constructed using Hisat2 v2.0.5, which was also used to align the paired-end clean reads to the reference genome. Genes were assigned to the Kyoto Encyclopedia of Genes and Genomes (KEGG) and the Gene Ontology (GO) databases for pathway and functional analyses. Raw reads were submitted to the Sequence Read Archive database under accession number PRJNA744053.

Differential expression analysis was performed using the DESeq2 R package (1.20.0). Thresholds including |fold changes| >2 and *p* < 0.05 were used to identify significantly differentially expressed genes (DEGs). DEG heatmaps were plotted using an online tool (https://www.omicshare.com/tools/, accessed on 29 September 2021).

## 3. Results

### 3.1. P. tricornutum Cell Abundance in Response to Toxicity Tests

**Figure 1:**
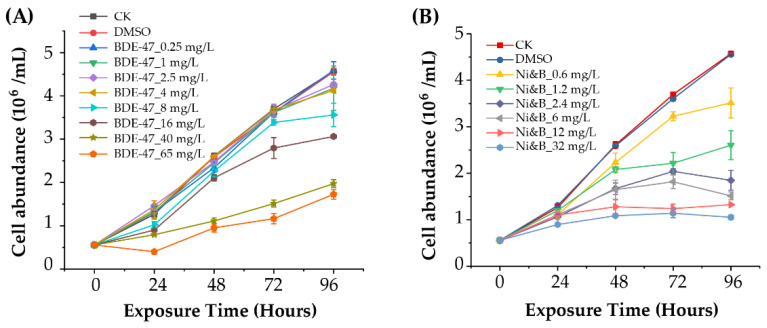
Cell abundance of *Phaeodactylum tricornutum* after individual BDE-47 treatment (**A**) and treatment with mixtures of BDE-47/nickel (**B**). Error bars indicate ± standard deviations (SD) of means. *p*-values are shown in Table S3.

No significant differences were observed in cell abundance in the BDE-47 toxicity tests at up to 8 mg/L of BDE-47 exposure over 72 h (*p* > 0.05) (Figure 1A). The cell abundance of BDE-47-treated cultures at concentrations of 16-65 mg/L was significantly lower than controls with the same exposure time (*p* < 0.001). After exposure to 65 mg/L BDE-47, cell abundance decreased at 24 h. Overall, the EC_50_-72 h of BDE-47 for *P. tricornutum* was 16.46 ± 0.93 mg/L. 

*P. tricornutum* growth was inhibited when exposed to BDE-47 and nickel. Further, the inhibitory effect on cell abundances gradually increased with increasing exposure concentration and time (Figure 1B). Significant inhibition of cell abundance was detected at 48, 72, and 96 h when using mixtures at a concentration > 0.6 mg/L (*p* < 0.01). The EC_50_-48 h and EC_50_-72 h of the BDE-47 and nickel mixtures for *P. tricornutum* were 3.68 ± 0.11 and 1.35 ± 0.06 mg/L, respectively.

### 3.2. Photosynthetic Efficiency Response

**Figure 2:**
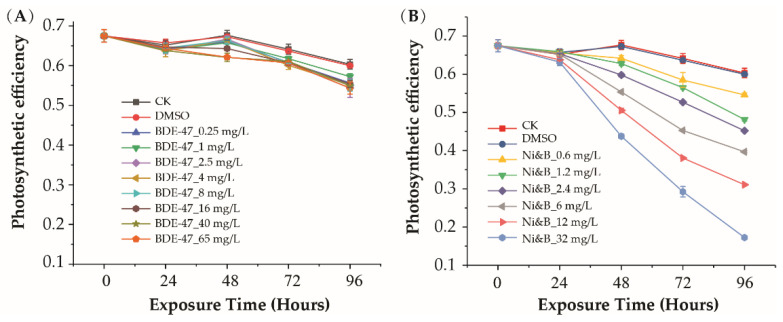
Photosynthetic efficiency of *P. tricornutum* after individual BDE-47 treatment (**A**) and treatment with mixtures of BDE-47/nickel (**B**). Error bars indicate ± SD. *p*-values are shown in Table S4.

The photosynthetic efficiency of *P. tricornutum* under different toxin treatments is shown in Figure 2A. BDE-47 exposure caused slight decreases in *P. tricornutum* photosynthetic efficiency. However, significant inhibitory effects were only detected at very high concentrations of BDE-47 (>8 mg/L). Photosynthetic efficiency was still relatively high in the 65 mg/L BDE-47 treatment.

Significant differences were not observed after exposure to mixtures of BDE-47 and nickel over 24 h. However, exposure to the mixtures strongly inhibited photosynthesis after 48 h of exposure. Photosynthetic efficiency gradually decreased with increasing exposure time and mixture concentrations (Figure 2B).

### 3.3. Reactive Oxidative Species (ROS) Production

**Figure 3:**
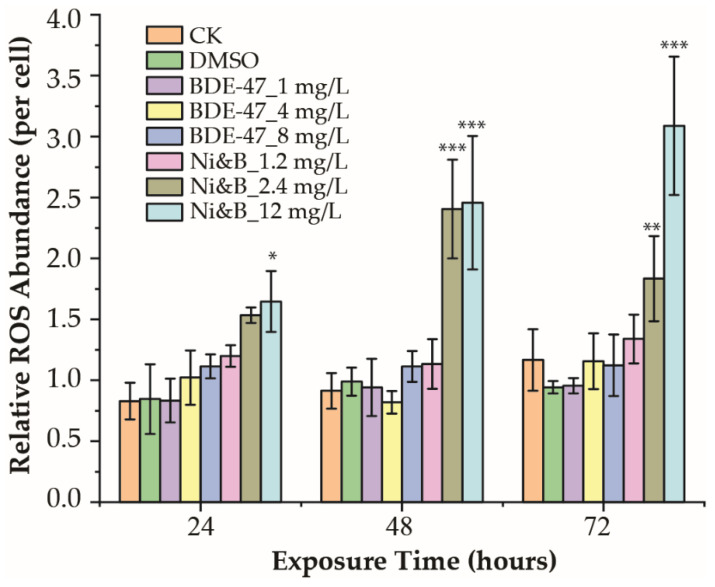
Relative ROS production of *P. tricornutum* after individual BDE-47 treatment and treatment with mixtures of BDE-47/nickel. *** *p* < 0.001; ** *p* < 0.01, and * *p* < 0.05. Error bars indicate ± SD. *p*-values are shown in Table S5.

ROS production levels over 72 h are shown in Figure 3. ROS production was induced by combinations of BDE-47 and nickel, although increases in ROS levels were not detected in the BDE-47-treated samples. The individual nickel treatments also demonstrated significantly increased ROS production [16]. Exposure to the BDE-47 and nickel mixtures significantly increased ROS production over 24 h at an exposure level of 12 mg/L (Figure 3). The highest ROS production was observed with 12 mg/L of mixtures after 72 h of exposure (Figure 3).

### 3.4. Interactions of BDE-47 and Nickel in P. tricornutum

The toxicity mechanisms of nickel and BDE-47 on *P. tricornutum* cells are not yet fully understood. Consequently, the potential interactions of nickel and BDE-47 were evaluated using concentration addition (CA) and independent addition (IA) models with the MIXTOX tool [33]. The two pollutants’ EC50-72h values for nickel, BDE-47, and mixtures were used to evaluate the interaction patterns. The *a* value was identified as −4.5, indicating that the interaction between nickel and BDE-47 was synergistic rather than antagonistic concerning *P. tricornutum* toxicity.

### 3.5. P. tricornutum Genes Differentially Expressed after Exposure to Mixtures of Nickel and BDE-47

A total of 565 and 325 genes were upregulated and downregulated after 48 h of exposure to nickel and BDE-47, respectively (i.e., those exhibiting |fold changes| > 2 and *p* < 0.05). After 72 h of exposure, these DEG numbers increased to 841 and 435 upregulated and downregulated genes, respectively (Figures S1 and S2).

**Figure 4:**
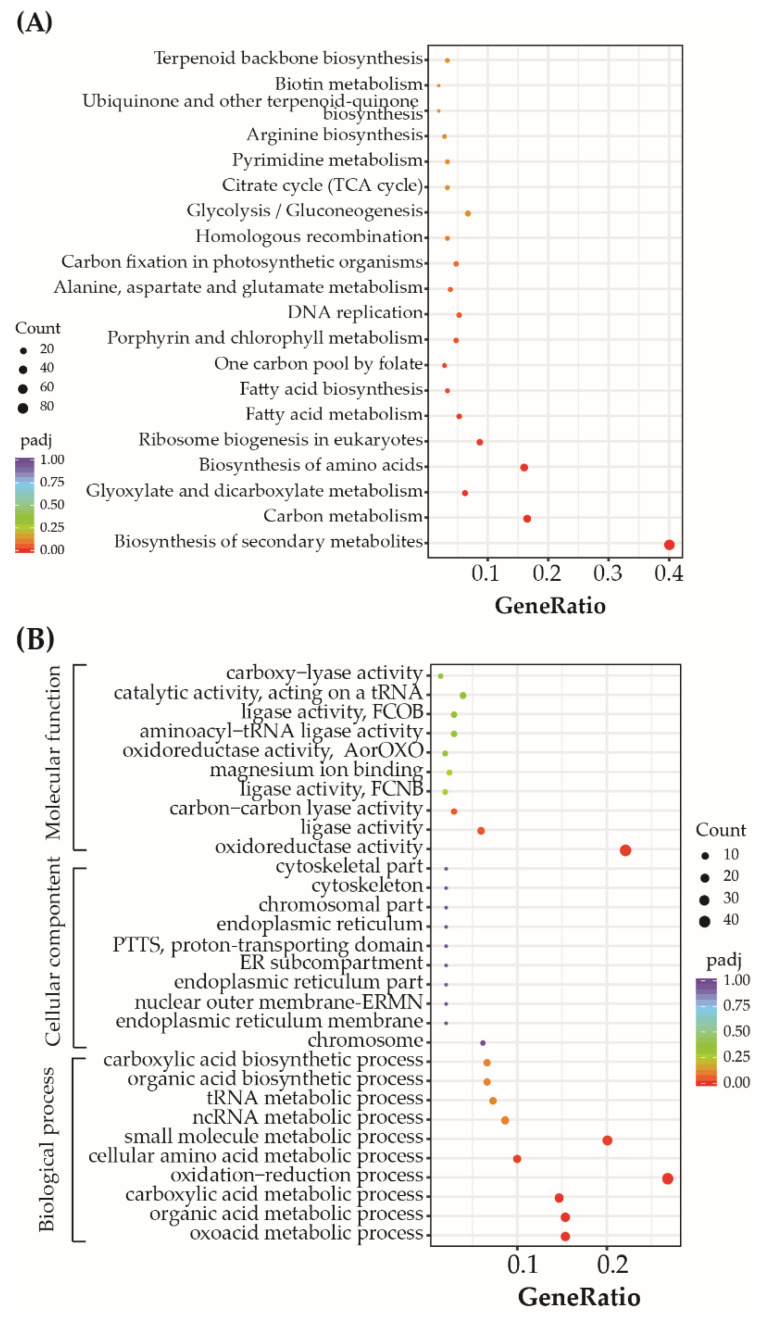
The 20 most abundant Kyoto Encyclopedia of Genes and Genomes pathways (**A**) and 10 most abundant Gene Ontology classifications (**B**) of differentially expressed genes after exposure to mixtures of BDE-47 and nickel treatments. Nuclear outer membrane-endoplasmic reticulum membrane network: ERMN; endoplasmic reticulum: ER; proton-transporting two-sector ATPase complex, proton-transporting domain: PTTS; ligase activity, forming carbon-nitrogen bonds: FCNB; ligase activity, forming carbon-oxygen bonds: FCOB; oxidoreductase activity, acting on the aldehyde or oxo group of donors: AorOXO.

Among the DEGs, 532 genes defined as core genes were upregulated or downregulated (|fold changes|>2, *p* < 0.05) after exposure to the mixtures for 48 h and 72 h (Figure S3). KEGG pathway analysis (Figure 4) of the core genes revealed that many of the DEGs were involved in the biosynthesis of secondary metabolites, carbon metabolism, biosynthesis of amino acids, glyoxylate and dicarboxylate metabolism, ribosome biogenesis in eukaryotes, fatty acid metabolism, fatty acid biosynthesis, and porphyrin and chlorophyll metabolism. Further, enrichment GO analysis of the core genes revealed many were involved in oxoacid metabolism, organic acid metabolism, carboxylic acid metabolism, oxidation-reduction reactions, cellular amino acid metabolism, and small molecule metabolism. In addition, oxidoreductase activity and ligase activity were significantly enriched GO terms involved in molecular functions.

### 3.6. DEGs Related to Photosynthesis and Nitrogen Metabolism

**Figure 5:**
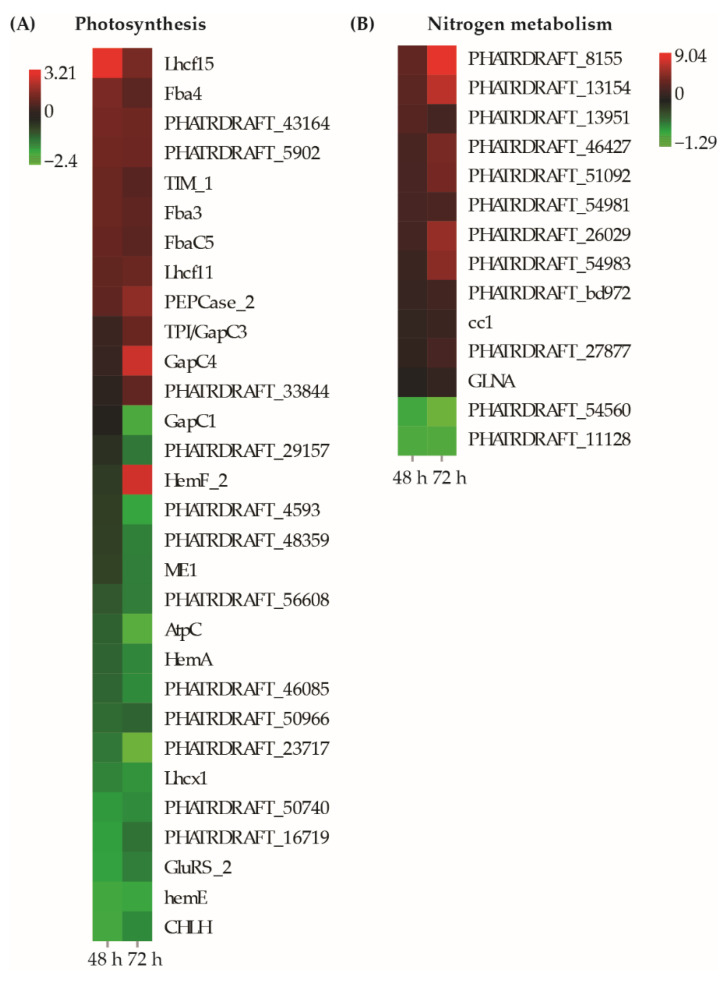
Heatmap of differentially expressed genes involved in photosynthesis and nitrogen metabolism that responded to exposure to mixtures of BDE-47 and nickel. Only genes with |log2 fold| changes > 2 and *p* < 0.05 after 48 h and/or 72 h are shown.

At least 31 genes related to photosynthetic metabolism were responsive to exposure to mixtures of BDE-47 and nickel (Figure 5A, Table S6). Among these, *HemA*, *UbiA*, *Aminotran_3*, *PHATRDRAFT_50740* (magnesium chelates), *hemE*, *GluRS_2*, and *CHLH*, which are involved in porphyrin and chlorophyll metabolism, were downregulated due to exposure to a mixture of BDE-47 and nickel. The photosystem II gene *PsbP* did not exhibit significant changes in expression after 48 h of exposure but decreased expression after 72 h. Most differentially expressed photosynthesis-related genes involved in carbon fixation were upregulated, including *Fba4*, *Fba3*, *FbaC5*, *TIM_1*, *PEPCase_2*, *TPI/GapC3*, *GapC4*, and *GapC1*. The antenna protein-encoding genes *Lhcf15* and *Lhcf11* exhibited increased expression due to exposure to the mixtures, while *Lhcx1* was downregulated after exposure to mixtures between 48 and 72 h. These results indicate that photosynthetic processes display different responses due to exposures to mixtures of BDE-47 and nickel, although the overall photosynthetic balance was disturbed.

A total of 14 DEGs were involved in nitrogen metabolism (Figure 5B, Table S7). Among these, 12 genes exhibited increased expression after exposure to mixtures of BDE-47 and nickel. *PHATRDRAFT_8155* (nitrite reductase), *PHATRDRAFT_13154* (Pyr_redox_2), *PHATRDRAFT_26029* (MFS_1), and *PHATRDRAFT_54983* (FAD_binding_6) exhibited very high expression levels with log2-fold changes >5 (Figure 5B, Table S7). Thus, nitrogen metabolism might be enhanced by exposure to mixtures of BDE-47 and nickel.

### 3.7. DEGs Related to Oxidation-Reduction Processes

**Figure 6:**
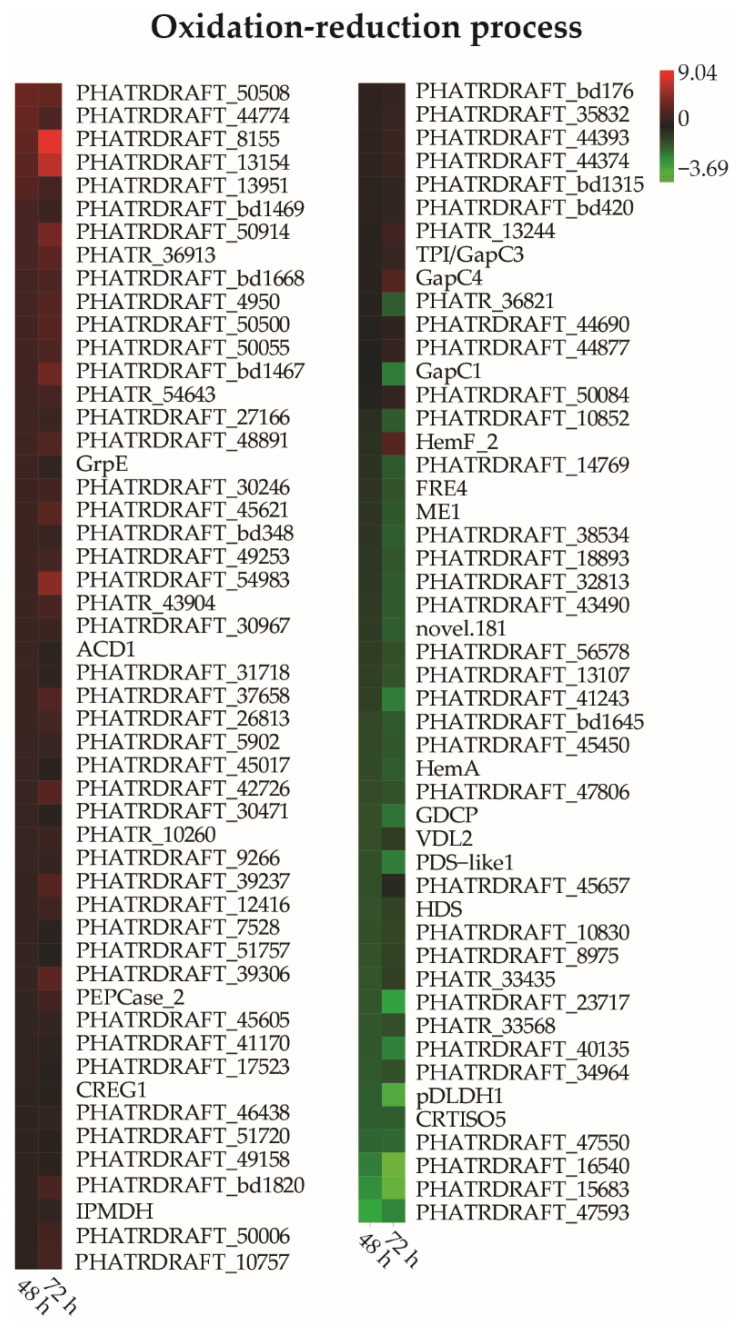
Heatmap of differentially expressed genes involved in oxidation-reduction processes that responded to exposure to mixtures of BDE-47 and nickel. Only genes with |log2 fold| changes > 2 and *p* < 0.05 after 48 h and/or 72 h are shown.

Many genes involved in oxidation-reduction processes were identified as DEGs (Figure 6, Table S8), indicating that the cellular redox balance was disturbed by exposure to the pollutant mixtures, consistent with the ROS measurements. Among the differentially expressed oxidation-reduction-related genes, some antioxidant genes exhibited increased expression. The most upregulated genes included a thioredoxin-like gene with log2 fold changes of 3.73 and 3.38 upregulated expression after 48 and 72 h, respectively. Glutathione *S*-transferase (*PHATRDRAFT_37658*, *GST*) was also upregulated due to exposure to the mixtures, with upregulation by 1.47 and 2.59 log2 fold changes after 48 and 72 h exposure, respectively.

## 4. Discussion

The toxic effects of nickel by itself on the diatom *P. tricornutum* have been evaluated in our previous studies. Specifically, nickel exposure exhibits an EC_50_-72 h value of 2.48 ± 0.33 mg/L and leads to ROS production and decreased photosynthesis [16]. In addition, the toxic effects of BDE-47 on *P. tricornutum* have also been reported [24]. Previous studies have indicated that nickel and BDE-47 can cause toxic effects on the diatom *P. tricornutum* by inhibiting cell growth and affecting photosynthesis [24]. However, the present study is the first to investigate the combined toxicity of BDE-47 and nickel on microalgae. 

BDE-47 and nickel exhibited synergistic interactions, with enhanced toxicity when mixed. Photosynthetic efficiency was strongly inhibited in *P. tricornutum* due to exposure to mixtures of BDE-47 and nickel, while inhibitory effects increased with increasing exposure time. However, BDE-47 by itself only slightly inhibited the photosynthetic efficiency of *P. tricornutum*, while nickel has been shown to strongly inhibit the photosynthetic efficiency of *P. tricornutum* [16]. Other studies of microalgae have shown that BDE-47 inhibits the photosynthetic efficiency of *Skeletonema costatum* and *Alexandrium minutum*. However, BDE-47 also reduced photosynthetic efficiency within 24 h of exposure in *Dunaliella salina* and *Thalassiosira pseudonana*, followed by gradual recovery to normal levels within 120 h [26,27,28]. Several differentially expressed photosynthetic genes at the transcriptional level, including those involved in chlorophyll synthesis, antenna proteins, oxygen evolution, electron transport, and downstream carbon fixation, were identified as DEGs of *P. tricornutum* after individual BDE-47 exposure, with most being downregulated [24]. Further, BDE-47 also suppressed the expression of some genes involved in photosystem II and antenna proteins in the diatom *T. pseudonana* [25]. In the present study, several genes involved in photosynthesis components such as carbon fixation, chlorophyll synthesis, photosystem II, and antenna proteins exhibited differential expression after exposure to mixtures of BDE-47 and nickel. In addition, nickel might impair the photosystem’s oxygen-evolving complex (OEC) in *P. tricornutum*, leading to decreased photosynthetic efficiency [16]. These data indicate that photosynthetic complexes may be one of the toxic effect targets of BDE-47 in microalgae and that its toxic effects on *P. tricornutum* photosynthesis could be enhanced with additional nickel supplementation.

Increased ROS is an indicator that cells were exposed to oxidative stress. Exposure to mixtures of BDE-47 and nickel significantly induced oxidative stress in *P. tricornutum*. Transcriptomic analysis identified DEGs involved in oxidation-reduction processes, with some involved in nitrogen metabolism, photosynthesis, and amino acid metabolism. Among these genes, antioxidant-associated genes, including those encoding thioredoxin (*Thx*) and glutathione *S*-transferase (*GST*), exhibited increased expression, implying that they might be important for oxidative stress resistance after exposure to mixtures of BDE-47 and nickel. Specifically, Thx could be involved in oxidative damage mitigation [36]. Thx possesses a conserved redox site that supports intracellular redox homeostasis, reduces protein thiols, and is one of the most important components of the thioredoxin system [37,38]. Increased expression of Thx has also been detected in individual nickel treatments of *P. tricornutum* [16]. In addition, *GST* plays an important role in the detoxification and reduction of ROS in multiple stress processes [39]. BDE-47-induced *GST* expression has been detected in *T. pseudonana* [25], while *GST* has been identified as a target of Thx in *Arabidopsis* plants [40]. Increased expression of *Thx* and *GST* indicates that detoxification activities were activated to protect cells from oxidative stress due to exposures to mixtures of BDE-47 and nickel. Thus, *Thx* and *GST* might simultaneously protect *P. tricornutum* cells from oxidative stress.

Transcriptomic analysis revealed that exposure to mixtures of BDE-47 and nickel could affect genes involved in oxoacid metabolism, organic acid metabolism, carboxylic acid metabolism, and organic acid biosynthesis (Figure 4B). Organic acids, such as citric acid and malic acid, play important roles in detoxifying chemical stresses, including those from BDE-47 and nickel [41,42,43]. The regulated organic acid BDE-47 has been detected in *Oryza sativa* [42,43], while nickel is also known to modulate organic acid compositions in *Alyssum murale* [41]. Isocitrate lyase (encoded by *PHATRDRAFT_51088*) is an enzyme that produces succinic acid and exhibited increased expression with 1.95 and 1.65 log2-fold change values after 48 and 72 h exposure to nickel and BDE-47 mixtures in this study. Succinic acid is involved in abiotic stress resistance in some plants [41,44,45]. For example, additional succinic acid concentrations can alleviate aluminum toxicity in alfalfa [44], while exogenous succinic acid concentrations lead to the enhanced tolerance of lead by *Larix olgensis* [45]. These data suggest that some organic/oxo-/carboxylic acids might be involved in the stress responses of *P. tricornutum* to mixtures of BDE-47 and nickel. In addition, organic/oxo-/carboxylic acids could serve as electron donors via their involvement in the dehalogenation process. For example, exogenous organic/oxo-/carboxylic acid-related compounds can be used as carbon sources (e.g., formate, acetate, and lactate) that could enhance the degradation of BDE-47 by microorganisms under anaerobic conditions [46,47]. Some microalgae, such as *Chlorella*, have been shown to transform BDE-47 into the debrominated product of BDE-47 [48]. We consequently hypothesized that some organic/oxo-/carboxylic acids produced by microalgae could be electron donors and are involved in transforming BDE-47 within algal cells.

The biosynthesis of secondary metabolites (Table S9), amino acids, and fatty acids were also extensively affected by exposure to mixtures of BDE-47 and nickel. Many organic acids are precursors for fatty acids, amino acids, and secondary metabolites [49]. Similarly, some secondary metabolites, amino acids, and fatty acids exhibited increased concentrations after BDE-47 treatment of rice [42,43]. Moreover, the biosynthesis of amino acids and fatty acid synthesis pathways were also affected by the BDE-47 treatment of the diatom *T. pseudonana*. Likewise, individual nickel treatment of *P. tricornutum* led to differentially expressed biosynthesis of amino acid and fatty acid synthesis pathways [16]. These results indicate that exposure to mixtures of BDE-47 and nickel extensively affected the primary and secondary metabolite profiles of *P. tricornutum*.

Nitrogen metabolism-associated genes were also detected among the DEGs of this study following exposure to the mixture of pollutants. The most expressed nitrogen metabolism-associated gene was *PHATRDRAFT_8155* (nitrite reductase), which is involved in nitrate assimilation, although other nitrogen metabolism-associated genes were also highly up-expressed. The enhanced expression of nitrogen metabolism-related genes was most prevalent in the individual BDE-47-treated *T. pseudonana* and nickel-treated *P. tricornutum*. These results suggest that the nitrogen metabolism of microalgae is highly sensitive to BDE-47 and/or nickel pollution. In addition, mixtures of BDE-47 and nickel can also enhance toxicity effects on nitrogen metabolism relative to the effects from individual chemicals.

## 5. Conclusions

In conclusion, the results from this study indicate that combined mixtures of BDE-47 and nickel pollutants were more toxic to *P. tricornutum* cells than either of the chemicals individually. Modeling analysis indicated a synergistic interaction between the pollutants, resulting in increased toxicity to *P. tricornutum*. Specifically, mixtures of BDE-47 and nickel decreased cell abundances, inhibited photosynthetic efficiency, and induced ROS production. Transcriptomic analysis further revealed that the mixtures affected photosynthesis, nitrogen metabolism, primary metabolism, and secondary metabolism processes, ultimately leading to cell growth inhibition in the diatom *P. tricornutum*.

## Data Availability

Not applicable.

## Supplementary Materials

The following supporting information can be downloaded at: https://www.mdpi.com/article/10.3390/toxics10050211/s1, Figure S1: Volcano plot of differentially expressed genes under 2.5 mg/L mixture treatment for 48 h; Figure S2: Volcano plot of differentially expressed genes under 2.5 mg/L mixture treatment for 72 h; Figure S3: Venn diagram of differentially expressed genes under 2.5 mg/L mixture treatment for 48 and 72 h; Table S1: Chemical concentrations tested in this study; Table S2: Results of Levene statistic test to determine homoscedasticity in data and statistic method used in ANOVA; Table S3: The P-value of comparisons with Games–Howell of cell abundance; Table S4: The P-value of comparisons with Games–Howell of photosynthesis; Table S5: The P-value of comparisons with Games–Howell of ROS; Table S6: DEGs related to photosynthesis; Table S7: DEGs involved in nitrogen metabolism; Table S8: DEGs involved in oxidation-reduction process; Table S9: DEGs involved in biosynthesis of secondary metabolites.
