# Supplementary material for: Toxicity Effects of Combined Mixtures of BDE-47 and Nickel on the Microalgae Phaeodactylum tricornutum (Bacillariophyceae)
Source: Toxics. 2022 Apr 22;10(5):211. doi: 10.3390/toxics10050211 (PMC9143900; doi:10.3390/toxics10050211)
Supplement: Supplementary file 1 [file toxics-10-00211-s001.zip › Supplementary Figures.pdf]

toxics

Supplementary Materials: Toxicity Effects of Combined Mixtures of BDE-47 and Nickel on the Microalgae *Phaeodactylum tricornutum* (Bacillariophyceae)

Xiaolai Shi, Ruoyu Guo, Douding Lu, Pengbin Wang and Xinfeng Dai

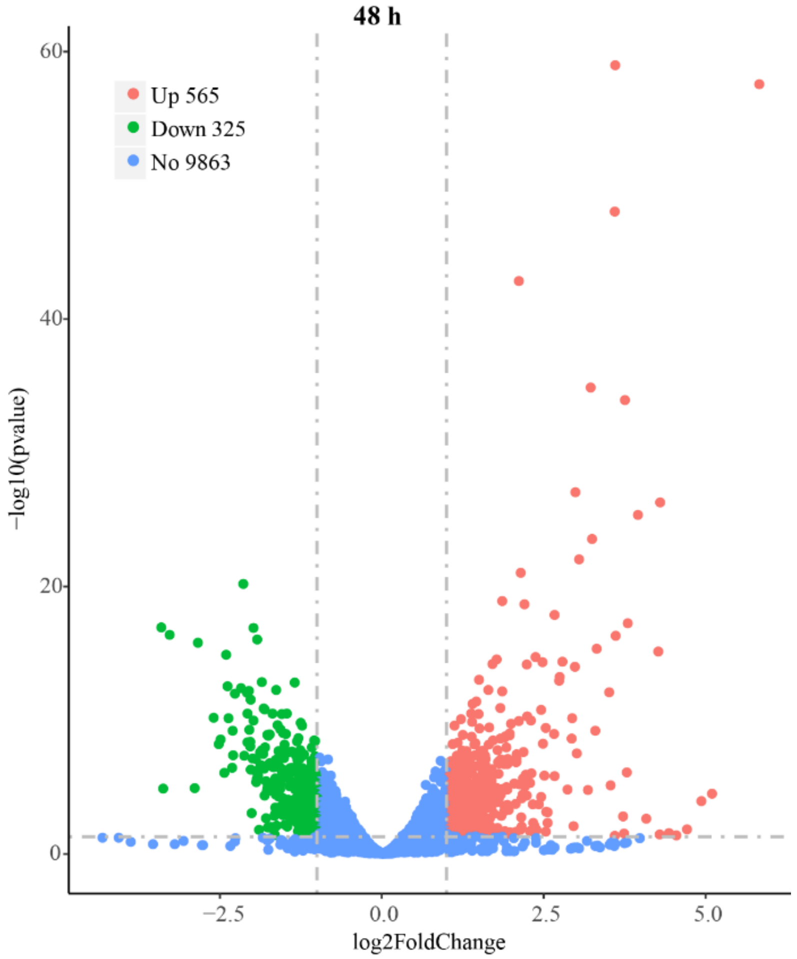

Figure S1. Volcano plot of differentially expressed genes under 2.5 mg/L mixtures treatment for 48 h.

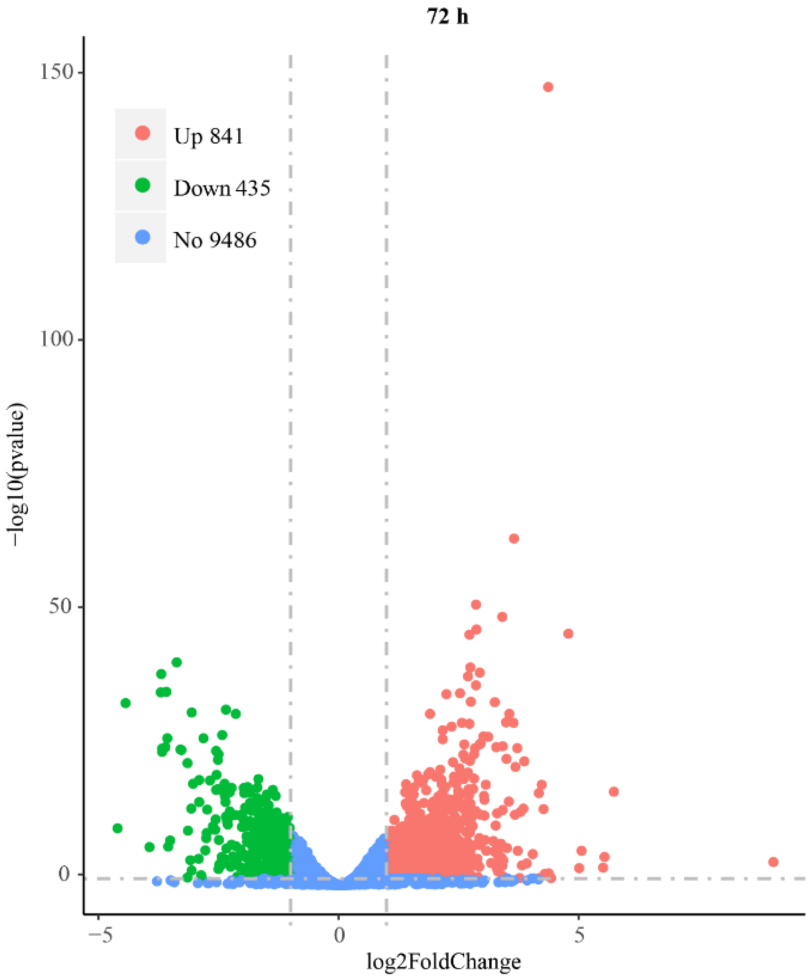

Figure S2. Volcano plot of differentially expressed genes under 2.5 mg/L mixtures treatment for 72 h.

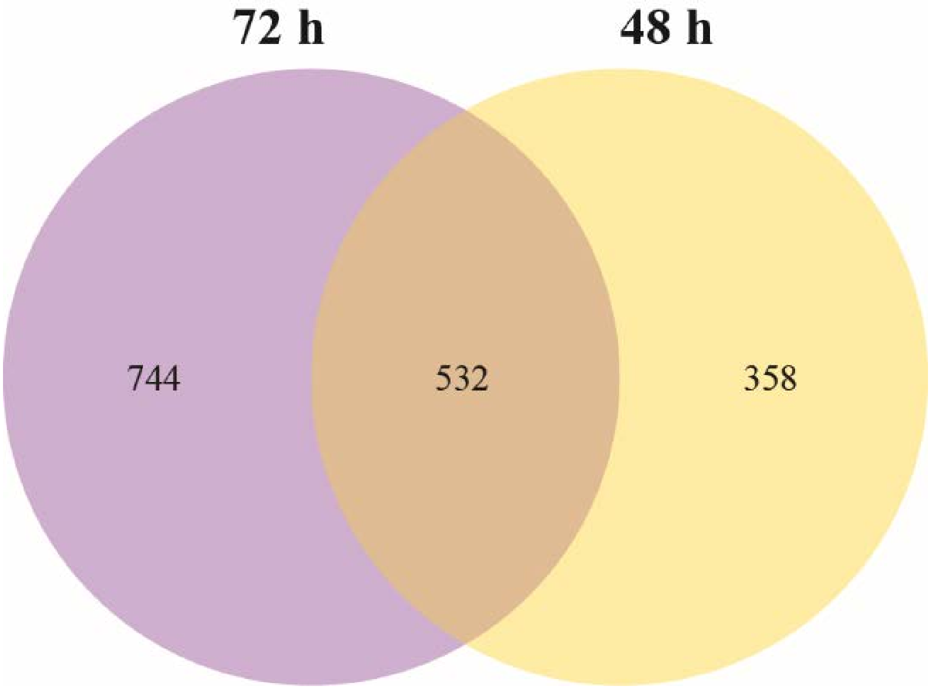

Figure S3. Venn diagram of differentially expressed genes under 2.5 mg/L mixtures treatment for 48 and 72 h.
